# When Learning Disturbs Memory – Temporal Profile of Retroactive Interference of Learning on Memory Formation

**DOI:** 10.3389/fpsyg.2018.00082

**Published:** 2018-02-16

**Authors:** Zrinka Sosic-Vasic, Katrin Hille, Julia Kröner, Manfred Spitzer, Jürgen Kornmeier

**Affiliations:** ^1^Department of Psychiatry and Psychotherapy III, University Clinic of Ulm, Ulm, Germany; ^2^Transfer Centre for Neuroscience and Learning, University of Ulm, Ulm, Germany; ^3^Institute for Frontier Areas of Psychology and Mental Health, Freiburg, Germany; ^4^Center for Mental Disorders, Medical Center, University of Freiburg, Freiburg, Germany; ^5^Faculty of Medicine, University of Freiburg, Freiburg, Germany

**Keywords:** retroactive interference, consolidation, memory, learning, decay

## Abstract

**Introduction:** Consolidation is defined as the time necessary for memory stabilization after learning. In the present study we focused on effects of interference during the first 12 consolidation minutes after learning. Participants had to learn a set of German – Japanese word pairs in an initial learning task and a different set of German – Japanese word pairs in a subsequent interference task. The interference task started in different experimental conditions at different time points (0, 3, 6, and 9 min) after the learning task and was followed by subsequent cued recall tests. In a control experiment the interference periods were replaced by rest periods without any interference.

**Results:** The interference task decreased memory performance by up to 20%, with negative effects at all interference time points and large variability between participants concerning both the time point and the size of maximal interference. Further, fast learners seem to be more affected by interference than slow learners.

**Discussion:** Our results indicate that the first 12 min after learning are highly important for memory consolidation, without a general pattern concerning the precise time point of maximal interference across individuals. This finding raises doubts about the generalized learning recipes and calls for individuality of learning schedules.

## Introduction

Learning and memory are very important ingredients in everyday life. Universities and schools are special with respect to learning and memory, because learning takes place in a very structured way over relatively long and intensive periods. Encoding and consolidation are two important initial steps during the formation of memory. In the typically very dense school and university schedules, encoding and consolidation from different learning units can temporally overlap and in the worst case interfere and thus negatively affect overall learning performance.

Theories of memory storage and forgetting have a long history of research with several examples of how an ongoing learning process can be affected by an interference with a secondary learning process. Such interference effects have already been reported in the “Kamin blocking effect” during classical conditioning, where an already learned association between an unconditional stimulus (US) and a conditional stimulus (CS) is weakened by the introduction of a secondary conditioning stimulus (Kamin, 1968).

A prominent role in the context of learning and forgetting has the interference theory: Forgetting is postulated to be caused by interference from distracting elements, either being presented prior to learning, which is known as “proactive interference” (Underwood, 1957) or after learning, during processes of consolidation, known as “retroactive interference” (Jenkins and Dallenbach, 1924; McGeoch, 1932). Numerous studies suggest that retroactive interference is a non-linear maximum function of the onset time point of post-learning distractors (e.g., Wixted, 2004). Thus, interference at a certain time point after learning may affect memory performance maximally. The important question is about the precise time course of such an influence. The first related studies were done by Müller and Pilzecker (1900). They found that people were less likely to recall cued items (nonsense syllables), if the intervening new stimuli were presented within the first 10 min after learning. Recall performance was better without interference. The authors concluded that the process of storing new memories needs time to stabilize during a process, which they called for the first time ever *consolidation*.

In the modern psychological literature research articles on interference effects during consolidation are surprisingly rare, with some notable exceptions (Lechner et al., 1999; Altmann and Gray, 2002; Lewandowsky et al., 2004, 2009; Wixted, 2004; Oberauer and Lewandowsky, 2008). These studies provide further evidence for the timing of the interference as a critical variable for consolidation. The broad range of relevant time scales ranging from seconds (Ecker et al., 2015) to months (Takashima et al., 2006) or even years (Smith and Squire, 2009) indicate that consolidation is a multistep process (Kornmeier and Sosic-Vasic, 2012; Wixted and Cai, 2013).

The issue of consolidation time scale(s) is substantially relevant to Cognitive Psychology in general, and to the Educational Psychology in particular, but hardly any research articles focused on the precise time course underlying retroactive interference of memory consolidation (for a review see Wixted and Cai, 2013).

Interest in consolidation as such, however, has been revived in the past decade within the field of cognitive neuro- and biological sciences (McGaugh, 1999, 2000; Dudai, 2004; Stickgold and Walker, 2005; Dewar et al., 2009; Squire et al., 2015) with some important new insights regarding the underlying neurobiological processes. Most important to our present work are findings from animal studies about time-dependent effects of interference on early consolidation. Examples are shuttle-box learning in goldfish and inhibitory avoidance learning in rats (Agranoff et al., 1966; Izquierdo et al., 1999, for a review see Dudai, 2004).

Only very limited human behavioral data is available about the time course of early consolidation with time scales in the range of minutes. Muellbacher et al. (2002) found that repetitive transcranial magnetic stimulation (rTMS) applied over the primary motor cortex within 15 min after learning in a skilled motor task, significantly disrupted the procedural memory performance. Cowan et al. (2004) focused on declarative memory. They presented word lists to patients with severe amnesia, and showed that retroactively interfering declarative stimuli within 10 min after learning impairs long-term memory consolidation, referring to the critical value of 10 min emphasized by Müller and Pilzecker (1900). Similar results were found by Della Sala et al. (2005) and Dewar et al. (2009).

The present study aims at direct investigation of the temporal profile of declarative memory consolidation on a time scale in the range of 12 min, referring to the seminal studies of Müller and Pilzecker (1900) and Cowan et al. (2004). Twelve minutes are in the range, highly relevant in school, since typical school lessons are in the range of 45 min and interference of one learning unit within such a lesson may have interfering effects on a previous unit. We thus focused on practical implications of retroactive interference effects on the time course of declarative memory within the first 12 min of consolidation in adolescents at school age during a vocabulary-learning task. Interference of memory formation can strongly depend on the similarity between the learned and the interference material (e.g., Pasternak and Greenlee, 2005). We thus decided to use lists of German–Japanese word pairs as both learning and interference material with the following focus:

(1) We expect a decay of memory performance within the first 12 min after learning, independent of the interference manipulation due to classical forgetting.
(2) Based on retroactive interference accounts, we further expect a non-linear impact of interference on memory performance with maximal interference at a certain interference time within these 12 min (e.g., Müller and Pilzecker, 1900; Della Sala et al., 2005; Dewar et al., 2009).

## Materials and Methods

### Participants

Thirty healthy German high school students (10 males and 20 females) were recruited from a German local higher school (“Gymnasium”). Five participants (four females, one male) missed at least one condition due to illness. We filled these gaps in the data matrix by the respective group means. The group had a mean age of 15.03 (years; months) ranging between 13.05 and 15.10 corresponding to normal 9th grade age within this school form. Participants were native German speakers without prior knowledge of Japanese language or culture, as the to-be-learned stimulus material was in Japanese language (see below).

The study was conducted according the ethical standards of the Declaration of Helsinki (Williams, 2008) and was in full accordance with the ethical, legal and regulatory norms and standards for research involving human participants in Germany as well as applicable international norms and standards. We took every precaution to protect the privacy of our participants and the confidentiality of their personal information. The study included only healthy normal participants and contained no invasive measurement and thus no danger to the participants’ health at any moment. During the experiment, participants simply read words from a computer screen and/or typed words with a computer keyboard. No experimental block lasted longer than 15 min and participants were allowed to pause or stop the experiment at any time if needed. We informed the participants at the beginning about the experimental details and the aim of the study. Also, student assent and parental informed consent to participate were obtained in writing prior to data collection.

In this study our participants did what they do nearly every day in school with the exception that we recorded their manual responses. Given this absolutely harmless procedure, we regarded a formal vote by the local ethics board as unnecessary.

### Experimental Task and Study Procedure

All participants underwent a verbal paired-associate learning task. We chose German–Japanese vocabulary learning as an instance of declarative memory formation in a realistic setting. In total, the paradigm contained two experiments (control and interference), each with four conditions. Both the *control* and the *interference experiments* were within-group designs with order of presentation counterbalanced across participants. There was a gap of at least 2 days between control and interference experiments for each single participant. Generally, we opted for a within-group design in order to reduce potential between-participant variability in memory performance and to save experimental time by reducing the number of experimental conditions (see below for explanation). Following each experiment, all participants answered questions related to their mental activities during the period between each initial learning session and the retrieval period, in order to control for possible rehearsal processes.

### Experimental Conditions

**FIGURE 1:**
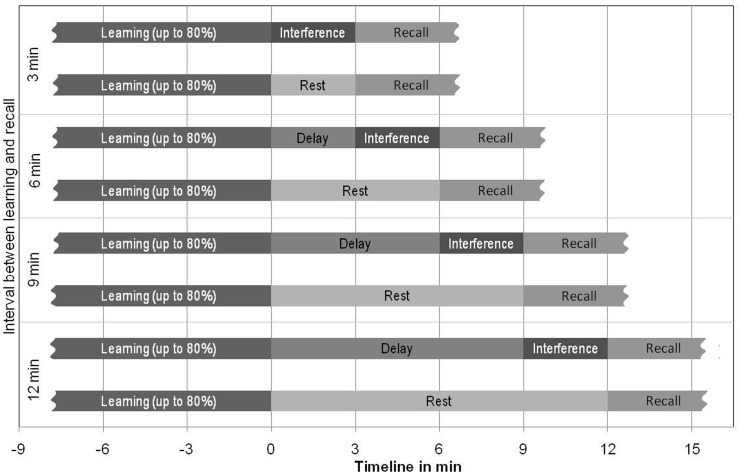
Paired-associate vocabulary learning paradigm. During the initial learning period (black regions on the left) participants learned twelve German–Japanese vocabulary pairs in each of the 2 × 6 = 12 experimental conditions until 80% recall performance. In separate experiments participants either performed an additional vocabulary-learning unit with 12 new word pairs (interference experiment) or they closed their eyes and relaxed (control experiment) during the subsequent retention interval (intermediate light and dark gray regions). Retention interval durations differed between experimental conditions. At the end of the retention intervals participants performed a cued recall test (dark gray regions on the right), where the German words from the initial learning period were presented and the Japanese words had to be recalled. The interference and control experiments were performed in a within-group design with the order of presentation counterbalanced between participants.

The interference experiment included three consecutive tasks (see **Figure 1**): an initial *learning* task, an intermediate *interferenc*e task introduced within a retention interval (=time between initial learning and final recall test) with four different delays of either 0, 3, 6, or 9 min after the initial learning task, and the concluding *recall* task to test memory performance immediately after the 3 min interference task. The control experiment was identical to the interference experiment with one exception: No interference task had to be executed in the retention interval. A sound presented via headphones announced the beginning of the recall interval. During the resting parts of the retention interval, participants were instructed to close their eyes and try to relax without holding specific thoughts. Also, participants were asked to refrain from repeating any of the previously learnt items.

During the *initial learning task*, participants had to learn one of eight different 12-pair German-Japanese vocabulary lists, each comprising common daily life words (one German, e.g., *Haus*, and its paired-associate Japanese Latin-transcribed translation, e.g., *yashikí*). The Japanese words were taken (*i*) for the sake of phonological novelty compared with the most European languages and (*ii*) because of providing a model of foreign language thesaurus learning in the school. The words were presented in parallel in the middle of the computer screen driven by the MATLAB Psychophysics Toolbox extensions for Microsoft Windows. Each word pair was presented for 7 s and followed by an inter-stimulus-time of 2 s. Participants were instructed to read the German word and then, with an emphasis, its corresponding Japanese paired-associate, and to try to remember the word pairings. Following a first presentation of all 12-word-pairs without any recall task, participants were presented with the German cue word and asked to write by keyboard the corresponding Japanese paired-associate. These keyboard responses to cue words at recall were recorded with the same software. For all conditions, the 12 associates from the list were repeatedly presented and tested in random order until a recall rate of 80% was reached.

Subsequent to this initial learning period we introduced the *intermediate interference task.* During this interference period, participants had to learn a new 12-pair German–Japanese vocabulary list. Stimulus presentation time and inter-stimulus-time were equivalent to the initial learning task. During first presentation of this new list no recall was required. Participants were instructed to read the German word and then, with an emphasis, its corresponding Japanese paired-associate, and to try to remember the word pairings. Following the first run of presentation of all 12 word pairs, participants were asked to respond to randomly and counterbalanced presented German cue words by keyboard writing of the corresponding Japanese associate. The duration of retroactive interference was set at 3 min. The interference task was scheduled immediately before the recall task (related to the initial learning list). Note that the interval between learning and recall in the different experimental conditions was the sum of the delay (0, 3, 6, or 9 min) and the duration of the interference task (3 min; as presented in the **Figure 1**).

The *concluding recall task* was administered immediately following the interference task (or following rest in the control experiment). At the concluding recall, the participants were shown German cue words from the list previously learned during the initial learning period and asked to produce their respective paired-associates. Again, items were presented in a randomized order to avoid possible item position effects. Participants were informed about the recall of these items.

In total, participants had to learn 144 German–Japanese word pairs across all conditions (interference and control experiments). To keep participants motivated for this rather time-consuming task, a voucher prize was awarded to the participant with the highest average score across selective reminding tests (i.e., cued recall) during the recall periods.

### Data Analyses

We calculated a generalized linear mixed model with a binomial family function and a logit link function, with the percentage of word pairs correctly recalled as variable and TIME and TREATMENT as factors.

We also calculated for each participant and interference time point the difference of the learning performance values between the interference experiment and the control experiment. We then isolated for each participant the maximal interference effect from these difference values and its location (i.e., the interference time point of this maximum within the 12 tested minutes). We further calculated the mean learning time within the initial learning task until achievement of the learning criterion (80%). The relation between mean learning time and the maximal interference effect was calculated with Pearson and Spearman Correlations. Raw data for all analyses are displayed in Supplementary Data Sheet 1.

## Results

**FIGURE 2:**
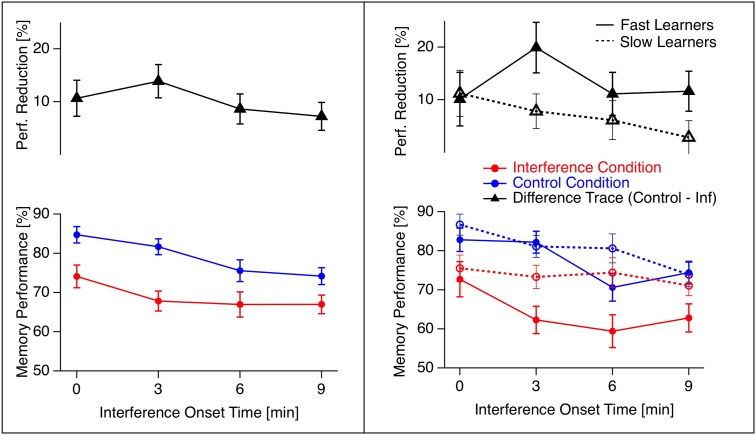
Temporal profile of retroactive interference (grand means). Mean memory performance in the interference experiment (red traces with circles) and the control experiment (blue traces with circles) together with the difference traces (control data minus interference data; black traces with triangles) across all participants **(left)** and for slow (dotted lines) and fast learners (continuous lines) separately **(right)**. Error bars indicate standard errors of the means. Standard errors for the difference traces result from the individual difference values of the participants. Interference obviously deteriorates learning performance (difference between red and blue traces) with larger effects for fast compared to slow learners (difference between black traces on the right).

The generalized linear mixed model revealed significant effects for learning success (intercept: *z* = 2.45, *p* = 0.01) and for the factor TREATMENT (*z* = -2.06, *p* = 0.04). No effect was found for the factor TIME nor for the interaction between TIME and TREATMENT. The mean results are depicted in **Figure 2**, left.

**FIGURE 3:**
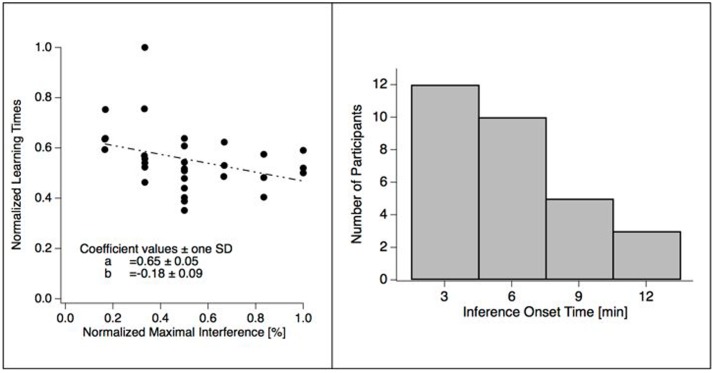
Temporal aspects of maximal interference. **(Left)** The scatter plot shows the maxima of individual interference as a function of learning time. Each dot represents one participant. The negative sloped dashed line represents a linear fit of the data and indicates a negative correlation between the two variables, i.e., fast learners are more susceptible to interference. **(Right)** The bar plot shows the numbers of participants with maximal interference values at each of the four interference time points after the initial learning sequence. Most participants peak at the first interference time point, however, location of the interference time point does not correlate with effect size.

Observation of individual data indicated a relation between the size of the maximal interference effect and the learning time. In *post hoc* explorative analysis we thus calculated correlations between the mean learning time and the maximal interference, and between learning time and the time of the maximal interference effect. We found a negative correlation between the maximal interference effect and learning time (*r*_Pearson_ = -0.35, *p* = 0.06; *r*_Spearman_ = -0.42, threshold = 0.36; see **Figure 3**, left, for a graphical illustration), but no significant effect between the time of the maximal interference effect and learning time.

We further divided the participants into two groups by median split of learning times and calculated a permutation test on maximal interference values between fast and slow learners. Fast learners showed a larger maximal interference effect than slow learners (*p* < 0.023, see also **Figure 2**, right).

## Discussion

In the present study we focused on negative effects of retroactive interference at different time points during the first 12 min of the memory consolidation period after learning of German–Japanese vocabulary word pairs.

We found that an interference task, where participants had to learn a different set of German–Japanese word pairs, reduced learning performance of the previous set by up to 20%. Our data indicate that the onset time of the interference content within the 12 min after learning is critical. For about 40% of the participants, interference was maximal within the first 3 min after learning. For about 25% of the participants’; interference was maximal between 6 and 12 min after learning. We further found that fast learners are more affected by the interference task than slow learners.

In conditional learning paradigms an already learned association is weakened by a later introduced secondary association (Kamin, 1968). On a more general level such a kind of interaction may also be regarded as a kind of interference effect. However, the effects we found in the present data, most probably appears earlier, during the consolidation of previously encoded information.

### Possible Confounds between Recall and Interference Time Points?

In the interference experiment we changed both the time point of the interference and the time point of the final recall concurrently. At a first sight it may seem difficult to decide which manipulation caused the effect. However, assuming linearity, the use of a full within-group design, enabled us to easily isolate the effect of interference time point from a possible effect of recall time point on an individual level. The significant effect of the factor TREATMENT (interference vs non-interference) can thus be related to the interference manipulation.

### Can Consolidation Interference be Temporally Resolved?

**FIGURE 4:**
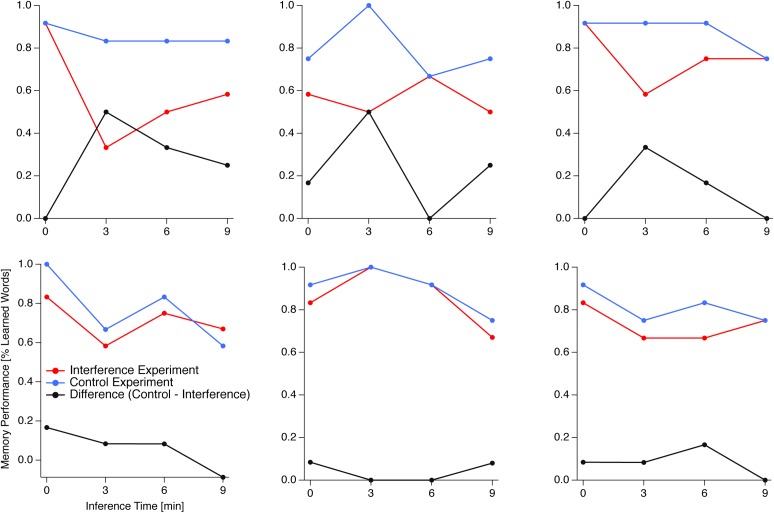
Temporal profile of retroactive interference (individual examples). Memory performance in the interference experiment (red traces) and the control experiment (blue traces) together with difference traces (control minus interference data; black traces) of six example participants. For five of the six participants the interference effect has a single maximum, which can be located at different interference time points. One participant (bottom middle) shows two peaks at the beginning and at about 9 min after the learning unit. Also effect sizes can differ considerably (compare top and bottom rows).

According to one of our hypotheses we expected the onset-time of the interference within the first 12 min after learning as a critical variable. Such an effect should be visible as an interaction between the factors TIME and TREATMENT. We did not find such an interaction, even though the grand means of the time-resolved differences between the interference and the control data indicate a peak at an interference time point starting 3 min after learning (black trace in **Figure 2**, left). The data show a large heterogeneity concerning the location of the time point of maximal interference as indicated by **Figure 3**, right, and by individual example data in **Figure 4**. A part of this inter-individual variability may be related to learning speed, as indicated by a moderate negative correlation between learning time and size of the maximal interference effect (see **Figure 3**, left).

In summary, the interference task has a clear detrimental effect on memory performance and fast learners seem to be more affected by this interference than slow learners. Most participants show one interference time point with a maximal interference effect. However, both the location and the size of this maximum vary between participants. Thus, a most critical onset-time of interference across participants within the first 12 min after encoding does not exist.

### Do We See a Retroactive or a Proactive Interference Effect?

The present data show the detrimental effect of the interference task on memory performance. But the question is, whether this interference effect is proactive, affecting the recall in the present paradigm, or whether it is retroactive, affecting the consolidation process.

In the proactive temporal-distinctiveness account the amount of interference is assumed to depend on the temporal proximity of the learning item and the interference item during encoding. The smaller the temporal distance between the two items, the more they interfere with each other and the larger is their competition during retrieval (Brown et al., 2007). The amount of interference is thus a question of proximity of the interfering material to the learning material. It should be largest if the interfering material occurs immediately after the learning material and it should decrease linearly with growing temporal distance between the learning and the interfering material. One could assume a kind of initial setting phase before interference between two successively perceived learning items starts, which would result in an inverted U-shape of memory performance as a function of interference time point. One could also assume some inter-individual variance concerning the duration of such a setting phase. However, proponents of temporal-distinctiveness approaches need to accept that such a setting phase may last for up to 12 min, which is rather long if we think about time constants for working memory in the seconds-range (Brown, 1958; Peterson and Peterson, 1959; Brown et al., 2007), which most probably is involved here. Further, this additional assumption makes a temporal-distinctiveness account a more complicated explanation than the retroactive interference account.

A linear decrease of the interference effect is not a necessary condition for the retroactive interference theory. Here it is simply the question at which time those consolidation processes start, that will be affected by a certain type of interference. Retroactive interference can happen already immediately or some seconds or minutes after the learning period (e.g., Wixted, 2004). The time point of consolidation interference is thus less restricted in the retroactive interference theory than in the temporal-distinctiveness theory. It may even depend on the interference content. Further, the retroactive interference approach does not rule out inter-individual variance concerning the time point of maximal interference, as found in the present study.

Our group-level analysis results show no effect for the factor TIME and thus no clear linear decrease of the interference effect. In particular, 40% of our participants show the largest interference effect at the first interference time point, i.e., immediately after the learning period, as predicted by the temporal-distinctiveness account. About 33% show maximal interference at the second interference time point, i.e., 3 min after the learning period and more than 25% of our participants show maximal interference between 6 and 12 min after the learning period (see **Figure 3** right and **Figure 4** for individual examples).

The large inter-individual variance concerning the time point of maximal interference can be interpreted as typical “experimental noise.” Alternatively, it may reflect different consolidation subtypes with different specific consolidation time constants. An interesting next step for future experiments may be a replication of the current experiment with a much larger sample size that would enable cluster analyses.

In summary, certain modifications of temporal-distinctiveness accounts may also allow for explanations of the large inter-individual variance. However, the retroactive interference theory is more plausible, given the principle of Occam’s razor. It may also be possible that both proactive and retroactive interference factors play a role and the future challenge will be to find the appropriate paradigm to disentangle such potential factors.

### Are Fast Learners More Sensitive to Interference?

Our data indicate that fast learners are more affected by our interference task than slow learners (*r*_Spearman_ = 0.42). Of course, this finding is not extraordinarily strong, and its based on a post-hoc analysis. It has thus to be considered with caution and needs to be replicated. In the case of a true effect, we want to offer a speculative explanation:

Assuming two participants being identical in every respect. During the learning task of the experiment the participants may be somehow unsure about the spelling of some learned Japanese words. One participant may guess correctly, the second incorrectly. The former may thus meet the 80% threshold and thus move on to the next step of the experiment, whereas the latter participant may repeat these words during the initial learning task. As a result, the one participant will be exposed more often to these word pairs and will thus learn them better than the other participant. His memory may thus be more resistant to subsequent interferences not because he is a slow learner, but because he had more exposure to the learning material. A number of such cases may explain the negative correlation on the group level.

### Practical Issues

Studies like the present one are always faced with a high-dimensional parameter space and experimenters need to decide – sometimes arbitrarily – about parameter ranges and/or values. Any such experiment thus only allows a view “through a keyhole.” In the current case it is rather unclear, whether interference onset times later than 12 min or a better temporal resolution of interference onset times would have shown larger interference effects. It is further unclear whether other interference material would be similar effective in affecting memory consolidation. Overall, the smallness of such typical “basic research keyholes” make practical conclusions difficult and thus invite for caution. However, this does not mean that any kind of conclusion is impossible or useless. In particular, we regard the following conclusions from the current experiment as relevant for praxis:

#### Consolidation of Memory Is in General Sensitive to Interference

After a certain learning unit, the system seems to need some time to internally stabilize (and transform) the learning content. The underlying processes seem to last at least for about 12 min, during which the system should not be disturbed. This time scale may be restricted to one of several consolidation steps and other critical time scales for consolidation probably exist as well (e.g., Kornmeier and Sosic-Vasic, 2012).

#### Need for Diversification

So far it is not entirely clear, what exactly does and what doesn’t disturb consolidation. We know from the present study and from the literature (e.g., Pasternak and Greenlee, 2005) that the same or a similar type of information has disturbing potential. Appropriate diversification of methods and contents within a school lesson or university lecture may thus reduce consolidation interference.

#### Allowing for Individualized Learning

The current data indicate that (a) there are specific interference onset times with maximal disturbing impact and (b) there is a huge inter-individual variability concerning these onset times. We have no general recipe concerning the maximally necessary consolidation time. Learners should thus be sensitive about their own learning time constants and teachers about their students’ learning time constants and the individuality thereof. Be careful with applying general learning rules. Again, diversification is indispensable.

#### Extinction of Bad Memories

So far we have emphasized the negative aspects of interference effects on memory consolidation. However, one can easily imagine negative information that one would prefer not to memorize. In such a scenario interference effects on memory consolidation may be utilized to prevent such undesirable memories^1^.

## Conclusion

Our data provide evidence for retroactive interference during memory consolidation and thus extend knowledge about interference effects on the consolidation of declarative memory. Fast learners seem to be more affected by interference than slow learners. The most sensitive time window for interference within the focused 12 min of memory consolidation seems to vary strongly between individuals. The latter finding indicate the need for more individually chosen starting points for resting or topic change after learning, and should therefore find even more consideration in structuring instructional settings and the multi-methodical composition of school lessons.

The present study has focused on interference effects within the first 12 min after learning. Studies on the cellular and molecular mechanisms of learning indicate different steps of consolidation at different time scales. The spacing effect and testing effect literature supports these findings with behavioral data in humans (e.g., Kornmeier and Sosic-Vasic, 2012; Kornmeier et al., 2014). Future studies should thus focus on the temporal profile of interference effects at longer time scales.

## Author Contributions

ZS-V: contributed to study the idea, design, sample recruitment, study the conductance, discussion of results, and writing the paper. KH: contributed to study the idea, data analyses, and discussion of results. JuK: contributed in literature review, discussion of results, and paper editing. MS: contributed in study the idea and discussion of results. JüK: contributed in data analyses, discussion of results, and writing the paper. ZS-V and JüK: literature review.

## Conflict of Interest Statement

The authors declare that the research was conducted in the absence of any commercial or financial relationships that could be construed as a potential conflict of interest.

## Footnotes

1 We would like to thank one reviewer for bringing up this important point.
