# Supplementary material for: When Learning Disturbs Memory – Temporal Profile of Retroactive Interference of Learning on Memory Formation
Source: Front Psychol. 2018 Feb 16;9:82. doi: 10.3389/fpsyg.2018.00082 (PMC5820352; doi:10.3389/fpsyg.2018.00082)
Supplement: Supplementary file 1 [file Data_Sheet_1.PDF]

Legend

IF	Interference Experiment
C	Control Experiment
Mem	Memory Performance Data in % of the total number of word pairs (= 12)
LT	Learning Time Data in msec

Participants were associated with a number and a gender index: f = female; m = male

Participant	IF_0_Mem	IF_0_LT	C_0_Mem	C_0_LT	IF_3_Mem	IF_3_LT
1_f	91,667	3056,455	83,333	3843,900	100,000	3818,417
2_f	66,667	2667,375	91,667	3416,364	83,333	2463,000
3_f	91,667	2378,818	83,333	7483,100	100,000	3596,917
4_f	66,667	2891,125	66,667	3169,375	91,667	2348,455
5_f	100,000	3292,750			83,333	2883,000
6_f	100,000	2005,917	75,000	5969,667	66,667	2533,750
7_f	91,667	4608,636	75,000	3398,556	83,333	2832,300
8_f	75,000	3189,556	50,000	2889,143	91,667	3139,636
9_f	83,333	2935,800	100,000	2764,750	83,333	2553,200
10_f	100,000	4287,833	83,333	4783,500	66,667	1832,625
11_f	91,667	4552,818	50,000	2494,000	66,667	2671,375
12_f	75,000	2623,667	100,000	4636,417	91,667	3068,818
13_f	83,333	2994,800			83,333	3334,200
14_f	75,000	2821,333	83,333	2322,900	75,000	2221,000
15_f	75,000	2440,667	41,667	2519,800	75,000	1767,222
16_f	83,333	2192,300	83,333	2192,300	100,000	1953,500
17_f	91,667	2147,091	50,000	2546,833	91,667	4262,273
18_f	58,333	2606,857	75,000	4459,667	83,333	2522,500
19_f	91,667	3740,545	91,667	3780,909	91,667	3157,545
20_f	75,000	2919,889	58,333	5356,714	100,000	2448,417
21_m	100,000	8083,750	91,667	4732,364	75,000	4029,889
22_m	75,000	2067,889	66,667	3436,000	66,667	2880,500
23_m	83,333	2209,600	58,333	2008,667	75,000	1953,889
24_m	91,667	2607,364	91,667	2228,818	83,333	5001,900
25_m	91,667	2813,364	66,667	4353,625	83,333	3837,700
26_m	75,000	2896,222	75,000	2584,000	66,667	3896,125
27_m	75,000	6315,667	66,667	4745,000	75,000	4210,778
28_m	91,667	4361,727	83,333	2714,100	66,667	3311,750
29_m	100,000	2185,667	50,000	3803,000	75,000	4765,222
30_m	91,667	2993,364	83,333	5728,100	75,000	8415,222

Onset of the interference task after the end of the learning task in seconds
_0_
_3_
_6_
_9_

C_3_Mem	C_3_LT	IF_6_Mem	IF_6_LT	C_6_Mem	C_6_LT	IF_9_Mem
91,667	6312,636	83,333	3256,800	100,000	3366,667	91,667
75,000	4367,667	58,333	6550,000	50,000	3658,400	83,333
100,000	4490,250	91,667	2664,545	91,667	8759,273	75,000
66,667	2095,500	66,667	3160,875	83,333	2511,500	66,667
50,000	2467,167	83,333	2561,400	83,333	2407,700	83,333
83,333	2220,800	41,667	1811,400	41,667	4080,600	50,000
75,000	5863,556	100,000	2977,583	83,333	2111,500	91,667
66,667	3657,625	50,000	2452,500	41,667	2109,250	75,000
58,333	1918,571	58,333	1989,143	75,000	3005,667	75,000
58,333	3221,571	83,333	2725,100	75,000	2147,778	58,333
83,333	4419,500	91,667	3392,182	66,667	5226,143	50,000
58,333	3358,429	91,667	2508,091	58,333	4292,143	75,000
58,333	2074,000	100,000	3956,333	75,000	8869,333	58,333
50,000	1335,000	66,667	1693,625	41,667	2360,400	75,000
83,333	2692,500	75,000	1883,889	33,333	2585,750	66,667
83,333	5019,000	83,333	2727,500	83,333	3711,900	100,000
66,667	2572,250	75,000	3440,333	58,333	3307,143	83,333
58,333	2620,286	66,667	3746,500	66,667	4119,625	66,667
58,333	2552,714	91,667	3216,818	75,000	3595,111	75,000
50,000	2197,167	66,667	4067,500	66,667	2829,375	75,000
66,667	4648,000	91,667	5622,182	75,000	8415,444	83,333
66,667	1996,375	75,000	5245,667	75,000	2733,667	75,000
		58,333	2548,143	41,667	2096,000	75,000
33,333	2008,750	83,333	3344,400	50,000	3026,833	83,333
66,667	3704,125	83,333	2220,200	100,000	5224,833	66,667
66,667	4872,750	58,333	6185,286	50,000	2537,333	66,667
83,333	2265,200	66,667	2815,333	66,667	2993,125	66,667
66,667	2352,625	58,333	2611,571	66,667	4664,625	66,667
75,000	3386,000	83,333	3017,100	66,667	3293,500	91,667
66,667	2729,875	83,333	9364,800	66,667	3512,750	75,000

IF_9_LT	C_9_Mem	C_9_LT
2928,182	91,667	5429,273
3191,200	66,667	2229,375
4576,667		
7154,875	66,667	7154,875
2485,900	66,667	4562,875
1902,167	58,333	1538,857
3519,273	83,333	2617,900
2991,778	58,333	3590,286
1663,444	58,333	2634,286
3444,857		
3461,333	66,667	2601,222
2128,111	41,667	3984,000
3483,167	75,000	3546,778
2440,667	75,000	2440,667
2539,875	58,333	3835,571
2674,500	100,000	2758,917
2566,800	58,333	3340,667
3100,625	83,333	2761,600
3034,111	75,000	8175,556
2889,222	50,000	2403,333
10651,200	83,333	3966,400
2749,889	50,000	4409,667
3855,111	58,333	2307,571
2534,100	58,333	5327,571
1992,500	75,000	3088,889
3610,125	66,667	5274,714
1851,875	66,667	4564,625
2818,375	50,000	4278,000
3471,545	58,333	5695,571
3090,444	75,000	2059,222
